# Abnormal Cystic Tumor in a Patient with Hereditary Leiomyomatosis and Renal Cell Cancer Syndrome: Evidence of a Precursor Lesion?

**DOI:** 10.1155/2015/303872

**Published:** 2015-08-25

**Authors:** Benjamin T. Ristau, Sonal N. Kamat, Tatum V. Tarin

**Affiliations:** ^1^Deparment of Urology, University of Pittsburgh Medical Center, Pittsburgh, PA 15219, USA; ^2^Department of Pathology, University of Pittsburgh Medical Center, Pittsburgh, PA 15219, USA

## Abstract

The hereditary leiomyomatosis and renal cell cancer (HLRCC) association is a rare syndrome caused by mutation of the Kreb's cycle enzyme, fumarate hydratase (FH). It is characterized by unusually aggressive type 2 papillary renal cell histology. FH is responsible for catalyzing the conversion of fumarate to malate. Its absence leads to a state of “pseudohypoxia,” inducing hypoxia inducible factor 1*α* (HIF-1*α*) and leading to increased growth factor transcription (e.g., vascular endothelial growth factor, VEGF; glucose transporter 1, GLUT1). Ultimately, this results in tumorigenesis. We present a patient who was diagnosed with HLRCC and underwent bilateral nephrectomies. One of the nephrectomy specimens was notable for benign cystic lesions that stained positive immunohistochemically for succinated proteins, a finding only noted in FH-deficient cells. Thus, we posit a potential precursor lesion to type 2 papillary renal cell carcinoma in the HLRCC syndrome.

## 1. Introduction

Hereditary leiomyomatosis and renal cell cancer (HLRCC) syndrome is a rare autosomal dominant familial disorder, in which affected families demonstrate a predisposition to the formation of uterine leiomyomas and aggressive type 2 papillary renal cell carcinomas. Launonen and colleagues first described this syndrome as one in which young (33 to 48 years old) females developed solitary kidney cancers that had already metastasized at the time of diagnosis. Genetic mapping demonstrated a loss of the normal chromosome 1q [1]. The first histologic renal tumor description was rendered shortly thereafter by Kiuru and associates who detailed a “rare papillary histopathology, which appears to be characteristic for HLRCC.” The nuclei were described as large, Fuhrman grades 3-4 and with inclusion-like large eosinophilic nucleoli. Moderate mitoses were noted and apoptotic cell groups were abundant. Neither necrosis nor hemorrhage was noted [2]. Within a year, the proposed locus at chromosome 1q was further characterized to chromosome 1q42.3–q43 and found to be the gene encoding fumarate hydratase (FH) [3]. FH is a Kreb's cycle enzyme that catalyzes the conversion of fumarate to malate. The mechanism of kidney cancer formation due to abnormal FH is related to an aberrant pseudohypoxic drive. Several authors have demonstrated accumulation of hypoxia inducible factor 1*α* (HIF-1*α*) and HIF-2*α* within HLRCC tumors leading to increased vascular density in uterine leiomyomas as well as an increase in glucose transporter 1 (GLUT1) in HLRCC kidney tumors [4, 5]. Thus, the mechanism of oncogenesis appears to be related to a metabolic shift from oxidative phosphorylation to aerobic glycolysis generated by a Kreb's cycle defect (i.e., Warburg effect), which stabilizes HIF-1*α* leading to increased expression of vascular endothelial growth factor (VEGF) and GLUT1 to provide substrates for rapid growth [6].

Bardella and colleagues were the first to demonstrate that aberrant succination of proteins in FH-deficient mice and HLRCC patients was a robust marker of FH mutation status [7]. Recently, a group from Michigan reported the first rapid (“warm”) autopsy report of a patient with HLRCC and advanced metastatic renal cell carcinoma [8]. On extended immunohistochemical analysis of the primary kidney tumor, the authors found strong expression of PAX8, vimentin, CD10, and the HIF target GLUT1. Tumor cells also demonstrated abundant accumulation of aberrantly succinated proteins (S-(2-succinyl)cysteine, 2SC) and p53. The authors conclude that this technique may enable better investigation of new and different aspects of tumorigenesis and metastasis.

We sought to apply these immunohistochemical techniques to our own case of HLRCC in order to better understand tumor development and biology in this rare entity.

## 2. Case Presentation

### 2.1. Clinical Case

**Figure 1:**
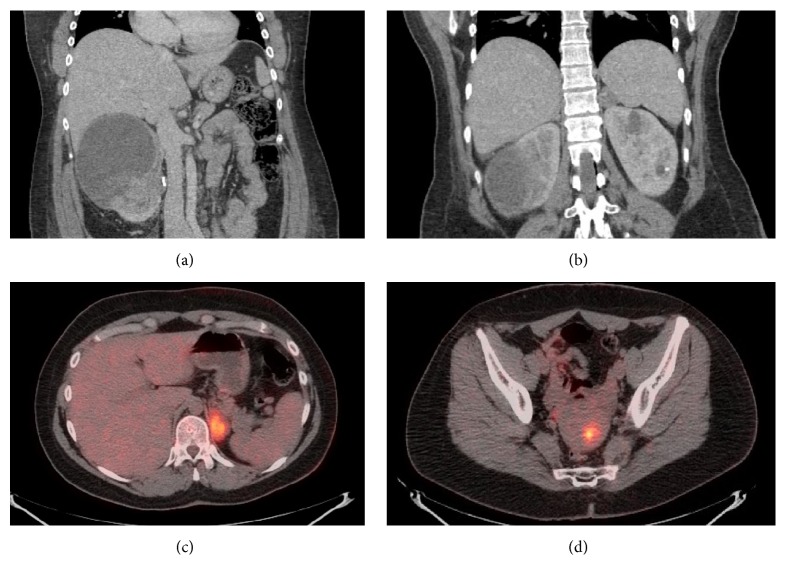
(a) CT representation of the right papillary type 2 renal cell carcinoma. (b) CT representation of multiple cystic structures within the left kidney. (c) PET scan demonstrating FDG-avid left adrenal lesion. (d) PET scan demonstrating FDG-avid uterine focus, likely a leiomyoma.

**Figure 2:**
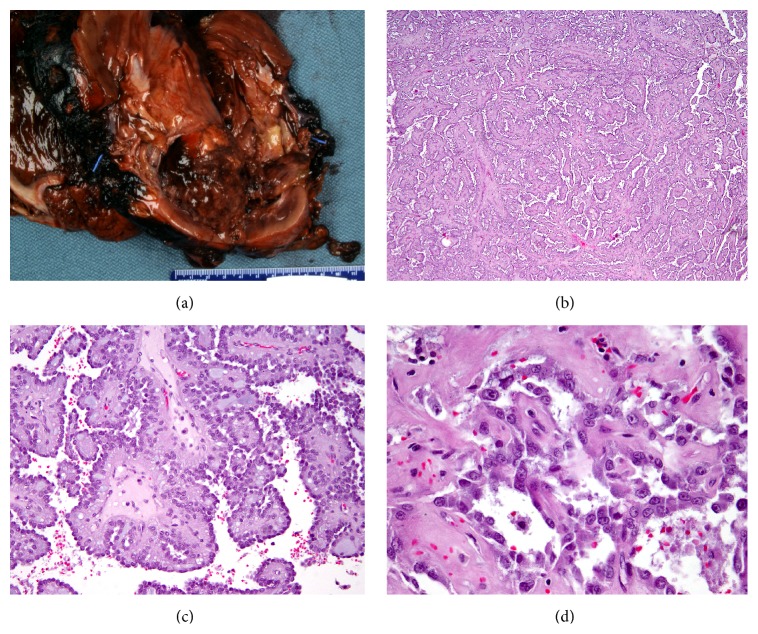
(a) Gross specimen of right radical nephrectomy demonstrating papillary renal cell carcinoma. (b) Low power H&E stained representation of papillary type 2 renal cell carcinoma. (c) H&E stained slide demonstrating papillary architecture. (d) H&E stained representtion of cytologic and nuclear atypia.

The patient is a 24-year-old woman at 35 weeks' gestational age that presented to the emergency department with right flank pain. An US demonstrated a 15 cm infiltrating and partially cystic right renal mass. She was induced and had an uneventful vaginal delivery. A CT scan was performed which confirmed an enhancing 15 cm right renal mass (Figure 1). She underwent right adrenal-sparing radical nephrectomy with paracaval lymphadenectomy. Final pathology from this surgery was reported as pT3aN1MX renal cell carcinoma of unclassified type (Figure 2). The histology was described as cells arranged in well-organized papillae with hobnail features, intraluminal blue secretions, villous-like architectural features, and a focus of bizarre nuclear atypia. FISH analysis was negative for trisomy 7-, trisomy 17-, and TFE3 translocation-associated renal cell carcinomas. Some tumor cells were noted to have very large eosinophilic nuclei with subtle perinuclear clearing. A suspicion for papillary type II histology was raised and the patient was referred for genetic testing. Indeed, a heterozygous 698G>A mutation was noted in the FH gene consistent with a diagnosis of HLRCC. Four months after her right nephrectomy, she underwent PET-CT imaging which demonstrated an FDG-avid left adrenal gland in addition to multiple septated left renal cysts (Figure 1). Given the known aggressive nature of HLRCC-associated type 2 papillary renal cell carcinomas, radical left nephrectomy with adrenalectomy and retroperitoneal lymph node dissection was performed and the specimen was submitted for pathologic analysis.

### 2.2. Pathologic Staining and Analysis

A left radical nephrectomy specimen and retroperitoneal lymph nodes were submitted for pathologic examination. Tissue underwent typical gross examination and sectioning. Formalin-fixed and paraffin-embedded tumor specimens were submitted in cassettes for microscopic examination. Hematoxylin and eosin staining was performed using a standard protocol on 20 tissue sections. Immunohistochemical stains for CK20, CA9, P504S, RCC, EMA, CK7, CD10, and vimentin were also performed on paraffin-embedded, formalin-fixed 4 *μ*m using standardized protocols with internal and external controls. Staining for aberrantly succinated proteins was accomplished using the 2SC antibody.

**Figure 3:**
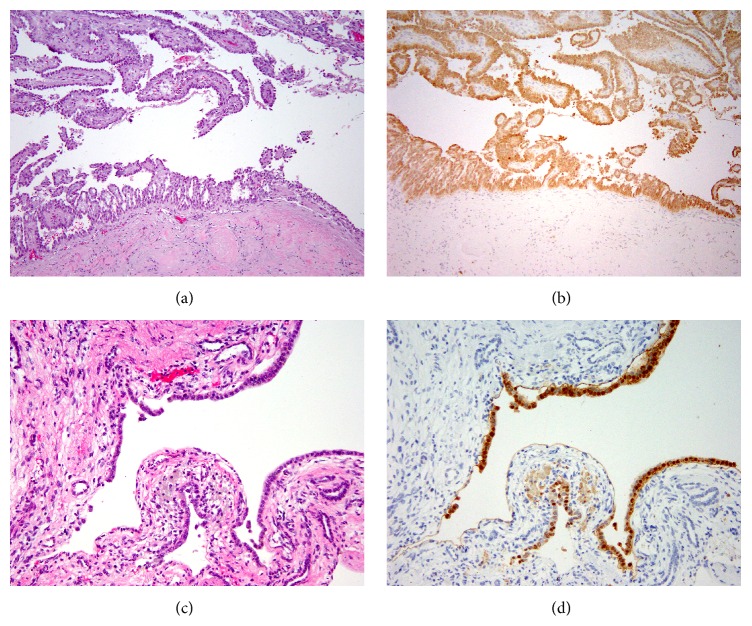
(a) H&E stained slide of a cystic component of the right confirmed papillary type 2 renal cell carcinoma. (b) 2SC stained slide of the right confirmed papillary type 2 renal cell carcinoma. (c) H&E slide of the left benign cystic renal lesion. (d) 2SC stained slide of left benign cystic renal lesion.

The gross description of the kidney delineates multiple thin-walled cysts filled with greenish, cloudy fluid in the upper and lower pole of the left kidney. No solid mass was identified. The tissue sections corroborate this with multiple simple cysts lines by a single cell layer with bland cytology. Some cyst-lining cells demonstrate variable amount of eosinophilic cytoplasm and enlarged nuclei with open chromatin and occasional prominent nucleoli. One section contains a single small papillary frond. In comparison with the right nephrectomy tumor specimen, subtle cytological similarity is noted, particularly in the cells lining the cystic spaces containing the papillary tumor (Figure 3). However, the cystic foci from the left kidney do not fulfill criteria for malignancy. 2SC staining of the benign cysts demonstrates profound uptake in the cells lining the cystic spaces similar to the 2SC staining pattern noted in the cystic components of the malignant right-sided type 2 papillary renal cell carcinoma.

## 3. Discussion

To our knowledge, this represents the first case of a histologically benign cystic renal lesion with evidence of FH mutation in a human patient with the HLRCC syndrome. The diagnosis is supported by findings of eosinophilic cytoplasm with enlarged nuclei and occasional prominent nucleoli. It is bolstered by the presence of positive immunohistochemical staining of the cyst wall lining with 2SC, indicating aberrant protein succination.

Frizzell and colleagues have demonstrated that excess fumarate in patients with diabetes reacts spontaneously with cysteine sulfhydryl groups to form a stable modification of proteins yielding, S-(2-succinyl)cysteine (2SC): a process called succination of protein [9]. Bardella and coworkers applied this concept to FH-associated neoplasia and demonstrated the presence of 2SC in murine FH-deficient renal cysts and in a retrospective series of 16 HLRCC tumors [7]. 2SC was not present in normal tissues and tumors not associated with HLRCC. Thus, 2SC was deemed to be an efficient and clinically useful marker of FH mutation in tumors.

Benign renal cysts have been previously described in humans with the HLRCC syndrome [10]. However, the finding of 2SC positive staining in the lining of otherwise benign renal cystic structures in humans with HLRCC is novel and suggests a potential preneoplastic lesion of the HLRCC syndrome-related renal cell carcinoma.

The pathogenesis of renal cyst formation in FH-deficient patients has been partially elucidated. In a murine model of HLRCC, Adam and colleagues have demonstrated that renal cyst formation is HIF-independent [11]. Their proposed mechanism involves the modification of cysteine residues by excess fumarate leading to an inability to suppress the nuclear factor (erythroid-derived-2)-like 2 (Nrf2) mediated antioxidant response pathway. The authors proposed that Nrf2 dysregulation may play a role in FH-associated cysts and tumors.

An alternative explanation is that benign cystic tumors in patients with FH-deficiency may not have encountered enough of the “pseudohypoxic” stabilization of HIF-1*α* mediated upregulation of VEGF and GLUT1 to complete the transformation into cancerous lesions. More research is needed into the pathogenesis of HLRCC-related type 2 papillary renal cell carcinomas to confirm these findings. If validated, such precursor lesions may represent a target for prevention of malignant transformation in this patient population.
